# Nordic Bioeconomy Pathways: Future narratives for assessment of water-related ecosystem services in agricultural and forest management

**DOI:** 10.1007/s13280-020-01389-7

**Published:** 2020-09-13

**Authors:** Jelena Rakovic, Martyn N. Futter, Katarina Kyllmar, Katri Rankinen, Marc I. Stutter, Jan Vermaat, Dennis Collentine

**Affiliations:** 1 Department of Soil and Environment, Swedish University of Agricultural Sciences (SLU), Uppsala, Sweden; 2 Department of Aquatic Sciences and Assessment, Swedish University of Agricultural Sciences (SLU), Uppsala, Sweden; 3 Finnish Environment Institute (SYKE), Helsinki, Finland; 4 James Hutton Institute, Aberdeen, Scotland, UK; 5 Norwegian University of Life Sciences, Faculty of Environmental Sciences and Natural Resource Management (NMBU-MINA), Ås, Norway

**Keywords:** Bioeconomy, Catchment modelling, Land use, Shared socioeconomic pathways, Storylines, Water quality

## Abstract

Further development of the bioeconomy, the substitution of bioresources for fossil resources, will lead to an increased pressure on land and water resources in both agriculture and forestry. It is important to study whether resultant changes in land management may in turn lead to impairment of water services. This paper describes the Nordic Bioeconomy Pathways (NBPs), a set of regional sectoral storylines nested within the global Shared Socioeconomic Pathways (SSP) framework developed to provide the BIOWATER research program with land management scenarios for projecting future developments to explore possible conflicts between land management changes and the Water Framework Directive (WFD). The NBPs are a set of narrative storylines capturing a range of plausible future trajectories for the Nordic bioeconomy until 2050 and that are fit for use within hydrological catchment modelling, ecosystem service studies and stakeholder dialogue about possible changes in agricultural and forestry management practices.

## Introduction

Development of the bioeconomy (bioresource-based economy) is on the policy agenda across Europe and considered an essential component of climate change mitigation and adaptation strategies (European Commission 2018). An increased reliance on bioresources can reduce societal dependence on fossil resources, thus contributing to a more circular economy. As part of the bioeconomy policy agenda, there are incentives to increase land-based biomass production of bioresource-based materials and fuels (Nordic Council of Ministers 2018). While bioeconomy developments are motivated by a desire to achieve environmental goals, it is not clear how this ambition in combination with recent incentives for sustainable intensification of agriculture to feed a growing world population (Rockstrom et al. 2017; Tilman et al. 2011) and the ensuing transformation in land cover and land management will affect the provision of ecosystem services.

The EU Water Framework Directive (WFD) dictates that activities leading to the degradation of water bodies from Good Ecological Status (GES) are either prohibited or subject to management restrictions (2000/60 EC). In the Nordic countries (Denmark, Norway, Sweden and Finland), these constraints may limit efforts to increase forest and agricultural biomass production. A concern over conflicts between WFD goals and the emerging land-based bioeconomy led to the creation of BIOWATER, a Nordic Centre of Excellence dedicated to examining the combined impacts of bioeconomy developments and climate change on land use, freshwater quality and water availability in the Nordic countries based on socioeconomic scenarios projecting possible future conditions in 2050 (https://biowater.info/).

There are two relevant factors to consider when investigating future developments in the Nordic bioeconomy. First, although agriculture is present to some degree in all Nordic countries; in Norway, Sweden and Finland there are large areas where current biophysical constraints dictate that these regions are primarily suitable for forestry and opportunities for agricultural expansion are limited. Second, Nordic economies currently trade openly in global markets and any shift in reliance on the land-based bioeconomy would be highly dependent on changes in the global economy. The second factor led to a decision to base future scenarios in BIOWATER on existing global socioeconomic future scenarios, while the first factor meant that the global scenarios also needed to be relevant for evaluating changes in Nordic land use and land management.

Generally, methods for developing socioeconomic scenarios can be divided into bottom-up or top-down approaches, or a combination of the two based on iterative processes. These approaches can be more or less participatory, involving collaboration with stakeholders at workshops or interviews (Absar and Preston 2015; Kok et al. 2007; Zurek and Henrichs 2007). Some scenarios are articulated as narrative storylines characterized by a set of elements considered as key drivers of change (Absar and Preston 2015; O’Neill et al. 2017). Conclusions derived from storylines then guide modelling of alternative future trajectories for the endpoints of concern (Riahi et al. 2017).

The climate change research community has followed a top-down approach in the development of a global scenario framework as a three-dimensional matrix: the shared socioeconomic pathways (SSPs), the representative concentration pathways (RCPs, greenhouse gas *concentration* trajectories) and a climate policy dimension (van Vuuren et al. 2013). In their ‘basic’ form, the SSPs are five storylines (SSP1–SSP5) that outline broad characteristics of global futures with different degrees of socioeconomic challenges for climate change mitigation and adaptation (O’Neill et al. 2017). The SSPs can be combined with biophysical climate scenarios (van Vuuren et al. 2011) to produce integrated scenarios.

The SSP developers wanted them to be useful for regional and sectoral analyses through downscaling and extending their storylines (O’Neill et al. 2017) and/or using quantified projections (Riahi et al. 2017). The SSPs are widely used in climate change impacts, adaptation, and vulnerability (IAV) research and include regional scale (e.g., Absar and Preston 2015; Kok et al. 2019) and sectoral (e.g., land use; Popp et al. 2017; Frame et al. 2018) extensions as well as extensions for water at global (Graham et al. 2018) and regional (Zandersen et al. 2019) scales.

As variation in socioeconomic futures presents at least as much complexity and uncertainty as geophysical projections (Vermaat et al. 2017), there is a pressing need to develop strategies for downscaling global socioeconomic scenarios to the local catchment scale that have the same rigor as strategies used for downscaling physical climate change scenarios. The most appropriate process for scenario development (e.g., top down–bottom up) and the degree of interconnectedness between scales (e.g., global–Nordic) are dependent on the issue being studied and the main purpose of the exercise—i.e., education, scientific exploration or decision support (Zurek and Henrichs 2007; Rounsevell and Metzger 2010).

Some recent studies have proposed guidelines for extending the SSPs to generalized European conditions (Kok et al. 2019), regions (Zandersen et al. 2019) and agriculture (Mitter et al. 2019). These three studies were embedded within broader multi-disciplinary research programs, which influenced the manner in which scenarios were developed. Kok et al. (2019) evolved from a European project, CLIMSAVE, with the objective to map previous scenario work to the SSP framework. They demonstrated how to operationalize extension of the SSPs and developed a set of European SSPs. Mitter et al. (2019) reported the development of a protocol for extending the SSPs to the European agricultural sector. They described their method for protocol development and outlined a series of steps for scenario extension. Zandersen et al. (2019) was primarily the result of collaboration among research programs with a focus on the Baltic Sea. They presented and operationalized a method for developing regional scenarios consistent with the SSPs, with the purpose to study future trends of Baltic Sea eutrophication, fisheries and marine life.

**Fig. 1:**
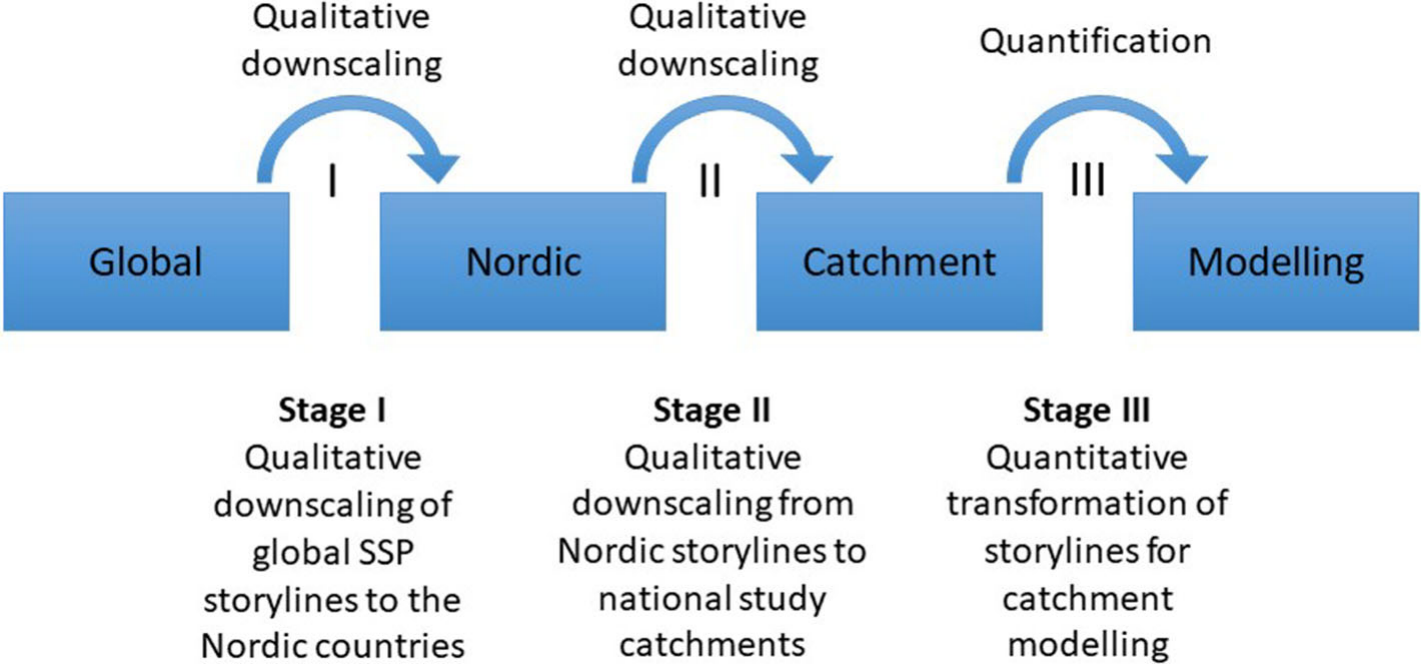
From global scenarios to catchment land use modelling

The downscaling and extension of the global SSPs into a set of storylines focused on the Nordic land-based bioeconomy—the Nordic Bioeconomy Pathways (NBPs)—was also embedded in a multidisciplinary research program, BIOWATER but was developed to meet particular needs in a particular context. The use of narratives to describe future pathways in this project was chosen as we believed this would allow for a greater role for qualitative interpretation and could also support BIOWATER researchers with a framework for providing high resolution data to be used in existing catchment-scale, process-based models. In contrast with the more generalized objectives of other studies (e.g., Kok et al. 2019; Mitter et al. 2019), the focus on meeting these particular needs led to a decision to work with a subset of the SSP elements. Figure 1 describes the structural flow of moving from the global (qualitative) to the local (quantitative) in the BIOWATER work. The work with Stage I in Fig. 1 has been completed and is described in this paper. Stage II which builds on the these results has also been completed through a series of national workshops in 2018 and 2019 and will be the subject of a forthcoming paper. Currently work with Stage III is in progress and expected to be completed in 2021.

In the first section below we present the methods we used to downscale and extend the global SSPs. Next, we describe and present the results: the NBP storylines. Finally, we analyze the NBPs and the development process in terms of the methodology used, compare our results with those from other relevant studies and explore the usefulness of the NBPs for other studies and the degree of success in achieving their objectives.

## Methodology

The development process was performed in five steps: (1) define objectives, (2) identify key elements (i.e., the main factors that together depict development of a Nordic land-based bioeconomy), (3) map elements across scales, (4) identify key regional and sectoral characteristics and (5) combine elements. This process is similar to the one used to construct the SSP narratives (Fig. 2 in O’Neill et al. 2017). However, we added two additional steps: “map elements across scales” and “identify key regional and sectoral characteristics” to facilitate downscaling the SSPs to a regional level (Nordic) and sectoral purpose (bioeconomy production in agriculture and forestry).

**Box 1:** NBP1: Sustainability first—Closing the loops

Societies around the world increasingly recognize the environmental, social and economic costs of disconnected, resource intensive production and consumption patterns. The development thus shifts to a more sustainable path, which respects perceived environmental boundaries and places human well-being ahead of economic growth. The changes in energy systems are directed towards renewables and high resource efficiency, coupled with consideration of the environmental footprint from the cradle to the grave. Along with the low resource intensive lifestyles, this leads to a low overall energy use. In the Nordic countries, the bioenergy share of energy use is relatively high and based on waste, residues and by-products. Policies in the bioeconomy sector are oriented towards development of sustainable and circular supply chains. Coupled to this there is a shift from linear to more circular and resource efficient land use, which include maintaining a balance between nutrient input and output. Land based production of biomass is regionally diverse, with locally adapted cropping systems designed to provide multiple benefits, including food, feed and fuels as well as delivery of other ecosystem services. Forestry moves towards continuous cover management systems and concerns about ecological impact leads to withdrawal from production on sensitive areas. The widespread environmental awareness of societies leads to low meat and low dairy diets. Considering this, animal husbandry moves towards small-scale farms that are adjacent to arable land, with grazing and foraging livestock. In this sustainability-oriented world, there are low challenges to climate change mitigation and low challenges to adaptation to the effects of climate changes.	

Openness was an important criterion of the narrative development process. The overall project objective (Step 1) was specified in the BIOWATER research proposal. Steps 2–5 were performed iteratively by two teams: a core group and a broader group of experts. The initial identification of key elements and mapping of the SSPs to the Nordic land-based bioeconomy were performed using expert judgment within the core group (the co-authors of this paper). Initial results were subsequently reviewed and developed further with an expert group comprising thirty BIOWATER researchers specializing in land, water and ecological management in key land use sectors across all Nordic countries during a workshop in 2018. Their comments and suggestions were incorporated into a new set of revised elements and storylines. The iterative process of dialog between core and broader groups continued until the first draft of the NBPs was finalized in December 2018.

### Define objectives

The primary study objective was the development of future narratives (for the year 2050) suitable for evaluating impacts on Nordic agriculture and forestry associated with a greater societal use of biological resources, i.e. land-based bioeconomy production. It was critical that these narratives could support subsequent analyses of the impacts of climate change, given its importance as policy driver for the greater reliance on renewable resources as substitutes for fossil resources.

### Identify key elements

We began by identifying the key elements that together depict development of a Nordic land-based bioeconomy (first column in Table 1). Identification was performed by first associating candidate NBP elements with SSP elements (second column in Table 1) and then by identifying regional and sectoral characteristics for each associated SSP element (columns 3 to 7 in Table 1) While some NBP elements were considered to be the primary societal drivers which would influence future outcomes for the Nordic bioeconomy, other elements were expected to determine limits on the outcomes, these are both regarded as key elements. For example, the “*bioeconomy policy orientation*” element is expected to drive development in a particular direction while “*population growth*” is expected to limit potential movement in a particular direction. It was not always possible to separate NBP elements into these two categories as some elements are both primary societal drivers and determinants of limits on outcomes. For example, the NBP element “*crop production”* may be driven by the “*environmental policy*” element where the latter may limit the extent to which the former can move.

**Table 1:** List of the scenario elements of the Nordic Bioeconomy Pathways (NBPs) and the corresponding driver scenario elements from the Shared Socio-economic Pathways (SSPs) (O’Neill et al. 2017) that were used to guide the development of each individual NBP element

NBP element	SSP driver element(s)	NBP1	NBP2	NBP3	NBP4	NBP5
Population growth		Relatively low	Medium	Low	Relatively low	Medium
	Population growth	Relatively low	Medium	Low	Low	Relatively low
	Migration	Medium	Medium	Low	Medium	High
Urbanization level		High	Medium	Relatively low	Medium	High
	Urbanization level	High	Medium	Low	Medium	High
Social equity		High	Medium	Low	Low	High
	Equity	High	Medium	Low	Medium	High
	Social cohesion	High	Medium	Low	Low, stratified	High
	Societal participation	High	Medium	Low	Low	High
Economic growth (per capita)		Medium	Medium, uneven	Slow	Medium	High
	Economic growth (per capita)	High in LICs, MICs; medium in HICs	Medium, uneven	Slow	Low in LICs, medium in other countries	High
Bioeconomy policy orientation		Toward sustainable production and consumption chains	Weak focus on sustainable production	Oriented toward self- sufficiency in the Nordic region	Toward the benefit of those with economic power	Toward new technology and free markets
	Policy orientation	Toward sustainable development	Weak focus on sustainability	Oriented toward security	Toward the benefit of the political and business elite	Toward development, free markets, human capital
Energy use and focus		Low; focus on renewables, footprint and resource efficiency	Medium; some investments in renewables, continued reliance on fossil fuels	High; expand domestic energy systems; some reliance on Nordic fossil fuels	Medium; diversified investments including efficiency and renewables, e.g. hydropower and wind power	High; no investments in low-carbon sources, heavy reliance on fossil resources
Bioenergy share and focus		Relatively high; novel technology, residue and by-product based biomass	Relatively low; but some investments in novel tech	Medium; mainly based on organic waste and forest harvest residues	Medium; reliance on imported bioresources	Low; limited incentives
	Tech development	Rapid	Medium, uneven	Slow	Rapid in high-tech economies and sectors; slow in others	Rapid
	Energy tech change	Directed away from fossil fuels, toward efficiency and renewables	Some investment in renewables but continued reliance on fossil fuels	Slow tech change, directed toward domestic energy sources	Diversified investments including efficiency and low-carbon sources	Directed toward fossil fuels; alternative sources not actively pursued
	Carbon intensity	Low	Medium	High in regions with large domestic fossil fuel resources	Low/medium	High
	Energy intensity	Low	Uneven, higher in LICs	High	Low/medium	High
Bioresource trade and systems		Moderate, circular i.e., focus on closing the loops, regionally diverse production	Moderate, linear supply chains, continuation of historical patterns	Strongly constrained, low- tech systems, focus on self- sufficiency	Moderate, linear, with increasing external costs	High, linear, high-tech regional specialization in biomass production
	International trade	Moderate	Moderate	Strongly constrained	Moderate	High, with regional specialization in production
	Globalization	Connected markets, regional production	Semi-open globalized economy	De-globalizing, regional security	Globally connected elites	Strongly globalized, increasingly connected
	Policy orientation	Toward sustainable development	Weak focus on sustainability	Oriented toward security	Toward the benefit of the political and business elite	Toward development, free markets, human capital
Crop production	Tech development	Rapid	Medium, uneven	Slow	Rapid in high-tech economies and sectors; slow in others	Rapid
		Diversification, locally adapted systems, focus on multifunctionality	Intensification with conventional approaches, moderate attempts to limit nutrient losses	Conventional input intensive, expansion where possible, whole removal of biomass	Conventional, with more precision agricultural approaches	Intensification of monocultures, resource- intensive high-tech farms
Forestry		Directed towards continuous cover with greater consideration of sensitive areas	Current Nordic model, i.e., dominance of even aged stands of coniferous trees	Current Nordic model but intensified management, low priority for environmental concerns	Current Nordic model	Current Nordic model, some intensification as Nordic timber export increases
	International trade	Moderate	Moderate	Strongly constrained	Moderate	High, with regional specialization in production
	Globalization	Connected markets, regional production	Semi-open globalized economy	De-globalizing, regional security	Globally connected elites	Strongly globalized, increasingly connected
	Environmental policy	Improved management of local and global issues; tighter regulation of pollutants	Concern for local pollutants but only moderate success in implementation	Low priority for environmental issues	Focus on local environment in MICs, HICs; little attention to vulnerable areas or global issues	Focus on local environment with *obvious benefits to well*-*being*, little concern with global problems
	Policy orientation	Toward sustainable development	Weak focus on sustainability	Oriented toward security	Toward the benefit of the political and business elite	Toward development, free markets, human capital
	Land use	Strong regulations to avoid environmental tradeoffs	Medium regulations lead to slow decline in the rate of deforestation	Hardly any regulation; continued deforestation due to competition over land and rapid expansion of agriculture	Highly regulated in MICs, HICs; largely unmanaged in LICs leading to tropical deforestation	Medium regulations lead to slow decline in the rate of deforestation
	Agriculture	Improvements in ag productivity; rapid diffusion of best practices	Medium pace of tech change in ag sector; entry barriers to ag markets reduced slowly	Low technology development, restricted trade	Ag productivity high for large scale industrial farming, low for small- scale farming	Highly managed, resource-intensive; rapid increase in productivity
Animal husbandry		Small-scale, grazing and foraging, adjacent to arable land for diversity and circularity	Medium-scale farms, some adjacent to arable land	Specialized, relatively large-scale, domestic feed	Medium-scale farms, some free range for elite comsumption	Specialized large-scale farms, domestic and imported feed
	Consumption and diet	Low growth in material consumption, low-meat diets, first in HICs	Material-intensive consumption, medium meat consumption	Material-intensive consumption	Elites: high consumption lifestyles; Rest: low consumption, low mobility	Materialism, status consumption, tourism, mobility, *meat*-*rich diets*
	Policy orientation	Toward sustainable development	Weak focus on sustainability	Oriented toward security	Toward the benefit of the political and business elite	Toward development, free markets, human capital

### Map elements across scales

Initial development of the NBPs was based on aligning them with the SSP framework, i.e. that higher scale scenarios provide strict boundary conditions (Zurek and Henrichs 2007). To ensure that the new narratives were consistent with the SSPs, the key NBP elements were associated with SSP elements with the latter used as a guide for mapping relationships from the global to the Nordic scale (Table 1). All SSP elements and their related assumptions were obtained from O’Neill et al. (2017) and from the supplementary information for Riahi et al. (2017). Not all SSP elements and assumptions were used to inform the NBPs in the mapping exercise. In our conceptual framework, an SSP element was omitted from the mapping if it satisfied either of the following two conditions: *i)* it was not considered a major driver of an NBP element or *ii)* it encompasses a research question for planned subsequent studies. For example, the SSP demographic elements (“*fertility”* and “*mortality”*) were not considered to be major drivers of the NBPs as they were expected to be the same over the study period (to 2050) regardless of the narrative and were therefore omitted. For example, O’Neill et al. (2017) made general statements under the element “*environment*” about whether environmental conditions are improving over time or not within each of the different SSPs. However, since our aim is to evaluate impacts on water resources and delivery of ecosystem services in subsequent research, we chose not to include assumptions about or predefine environmental outcomes in the NBPs. This approach enables future comparison between the environmental outcomes assumed under the SSPs as well as Nordic environmental outcomes that are a result of ecosystem service studies and catchment modelling conducted elsewhere in the BIOWATER project.

Some SSP elements map to multiple NBP elements (Table 1). These elements are repeated either individually or as a group. For example, the SSP element “*technological development”* is associated with the NBP elements “*energy use and focus”* and “*bioresource trade and systems*”. While the same group of SSP elements in the NBP element “*bioenergy share and focus”* which is identical with the NBP element “*energy use and focus”*. All of these mappings were performed so that we could extrapolate trends from the SSP assumptions relevant to our primary objective.

In some instances, addressing issues of increased reliance on agricultural and forest bioresources required expansion of elements beyond current reported studies. In Table 3 of O’Neill et al. (2017) the SSP element “*agriculture”* only includes general information, e.g., there is “low technology development and restricted trade” in SSP3 while in SSP5 there is a “highly managed, resource-intensive” agriculture with a “rapid increase in productivity”. Given the importance of agriculture in our objectives for narrative development we expanded this category into two separate NBP elements: “*crop production”* and “*animal husbandry*”. We then associated relevant SSP elements with each NBP element individually.

Forestry is the other major sector included in the NBPs. The SSPs do not contain information about forest management other than about deforestation, which is not a major issue in the Nordic countries. To extend the SSPs from a forestry perspective, we used key information from relevant SSP trends for the NBP element “*crop production”* since to a great extent the NBP “*forestry”* element is driven by similar forces. However, in Norway, Sweden and Finland there are significant biophysical limitations to the ability to transform forested land into agricultural production and institutional limitations precluding transformation of agricultural land to forests. In Denmark while there are fewer biophysical limitations, the institutional limitations are similar.

### Identify key regional and sectoral characteristics

In this step, we focused primarily on biophysical, institutional and cultural characteristics of the Nordic countries that may either amplify or diminish the global SSP drivers. For example, in the Nordic countries converting forests to agricultural production is not a significant factor for reasons mentioned earlier and because forestry is a long term investment. When agricultural land is afforested it is locked up for an extended period (50–100+ years) and as a result, landowners may be reluctant to convert based on short term factors. These characteristics serve to limit extensive expansion in either agriculture or forestry. Thus, more importance in the NBP narratives is placed on changes in production related to intensification, i.e., increasing/decreasing inputs and outputs per unit area. The high level of cooperation between the Nordic countries is another key regional characteristic. The historical degree of openness in the Nordic region means that while national policies are not necessarily coordinated, they do take into consideration the ease of movement (and long land and sea borders) which serves to limit any significant differentiation with respect to trade. Other limitations enter into the narratives through assumptions associated with each of the pathways documented below.

### Combine elements

The last step in NBP development was to construct storylines that are consistent with both regional and sectoral characteristics and the SSPs. Development of the NBP storylines used a back-casting approach similar to methods used to develop the SSP storylines (O’Neill et al. 2017). The starting point for extending the SSPs was to keep consistency across scales (global to local) with respect to assumptions made and to the degree of socioeconomic challenges to adaptation and mitigation. Thus, the NBPs are positioned in the same future outcome space as the SSPs (see Fig. 1 in O’Neill et al. (2017). Since our objective was to explore the potential future effects of changes in land cover and management on water quality and quantity, we wanted the global scenarios to accommodate extensions with a wide spread in land cover and management futures to enable addressing a broad range of possible future conditions in the Nordic countries. In certain cases, e.g., assumptions about environmental policy in SSP4 and SSP5, trends were described similarly for high-income countries (O’Neill et al. 2017). Data from the SSP database (https://tntcat.iiasa.ac.at/SspDb) for 2050 were also used for support. Moreover as noted above, SSP trends in land use (which focused on deforestation and agricultural expansion) were not applicable in the Nordic countries due to regional biophysical and institutional constraints. However, by combining other qualitative information, such as that international trade is “high, with regional specialization in production” (O’Neill et al. 2017) we obtained sufficient information to enable a spread in land cover and management futures.

## Results

The identification of key scenario elements and mapping of NBP elements to SSP drivers and the formulation of trends for each NBP element in Table 1 are the basis for a textual description for each of the pathways. These descriptions, the five NBP storylines (Boxes 1, 2, 3, 4, 5), are summarizations of the NBP element trends in Table 1. The storylines describe in a simple way the implications for the future Nordic bioeconomy in 2050 if global socioeconomic developments follow pathways similar to each of the corresponding SSPs. The text following each NBP below includes a short description of how the future development pathway for that particular NBP could plausibly emerge based on the associated trends in Table 1 and some of the differences between the pathways.

**Box 2:** NBP2: Conventional first—don’t rock the boat

This world follows typical recent historical patterns with uneven development and income growth. There is a concern for local pollutants but moderate success in policy implementation and slow progress in achieving the sustainable development goals. In the Nordic energy sector, some investments in renewable energy systems are made but society continues to rely on fossil fuels. Bioenergy is a relatively low share of total energy use although there are some investments in novel technology. In the bioeconomy sector, there is an overall weak focus on sustainability with continued dependence on disconnected (linear) supply chains from production of biomass to consumption. Within the agricultural sector, the emphasis is placed on intensification of production with conventional approaches, including moderate attempts to limit nutrient losses. Although overall consumption is material-intensive, there is a slight downward trend in meat consumption and a parallel trend to slightly less intensive livestock operations. Forest management follows the prevailing Nordic model, with a dominance of even aged stands. In this middle-of-the-road society there are moderate challenges to climate change mitigation and adaptation.

**Box 3:** NBP3: Self-sufficiency first—Building walls

The world is characterized by rising regional rivalry driven by growing nationalistic forces and the Nordic countries have become allies in a fragmented Europe. International trade is strongly constrained and policies are oriented towards security, while there is low priority for environmental issues. The importance of developing the Nordic bioeconomy therefore becomes a matter of regional security, placing self-sufficiency aims high up on the agenda. Energy consumption is high and prevailing Nordic energy systems and supplies such as hydropower and Norwegian oil are expanded. There is also a moderate rising trend in domestic bioenergy production, including biofuels mainly produced from organic waste and forest harvesting residues. Technology development is, however, slow in all sectors. Strategies for increased self-sufficiency of food, feed and bioenergy focus on intensifying conventional agricultural practices as well as expansion of arable land where possible. A rise in domestic meat production and meat rich diets are supported by more specialized and concentrated livestock operations. Nordic forest management is also intensified and there is a low priority for environmental considerations. Due to lack of international cooperation, low environmental awareness and material intensive consumption patterns there are high challenges to climate change mitigation and adaptation.

**Box 4:** NBP4: City first—Maintaining the divide

In a world with unequal investments in human development and rising differences in economic opportunity and political power, a gap widens across and within countries between a small affluent elite and underprivileged lower-income groups. Environmental policies are centered on local concerns with little attention to vulnerable areas or global issues. In the Nordic countries, segregation between societies in overlooked residential areas and more valued prosperous regions continues to lower societal cohesion. Rural areas that are not favorably situated for tourism are increasingly neglected because policy is oriented toward the benefit of those with economic power. Big corporations gradually take over the land-based bioeconomy sector at the expense of small-scale family farms and individual forest owners. Due to an uncertain fossil fuel market, there are diversified investments in the energy sector, including efficiency and renewables. The bioenergy share of energy use follows an upward trend facilitated by rising import of bioresources to the Nordic countries. In the forestry sector the current Nordic model prevails. Strategies in the agricultural sector are steered towards conventional crop production with more precision agricultural approaches, while animal husbandry is diversified. Due to some low carbon investments and a well-connected international political and business class there are low challenges to climate change mitigation. Challenges to adaptation to the effects of climate change are, however, high.

**Box 5:** NBP5: Growth first—Running on the treadmill

Spurred by high economic growth and rapid technological development, this society trusts that competitive markets, new technology and investments in human capital is the path to sustainable development. Regarding environmental policy, there is a focus on local issues with obvious benefits to human well-being, whereas global issues receive little attention. In this society, lifestyles are material intensive and diets are meat rich. The energy and resource use intensity is high and there is a heavy reliance on fossil resources. With increasingly connected global markets, biomass production moves towards more large-scale regionally specialized systems, also in the Nordic countries where intensification of forestry production systems is driven by rising timber export. There are however limited incentives to develop the bioenergy sector. In the agricultural sector, crop production systems move towards intensification of monocultures and resource intensive high-tech farms, while animal husbandry becomes more specialized and concentrated in large-scale farms. In this fossil fuel dependent society there are high challenges to climate change mitigation. However, a highly engineered infrastructure leads to low challenges to adaptation.	

### NBP1: Sustainability first—Closing the loops (Box 1)

This is the greenest of the five pathways. The underlying assumption is that while there will be increased substitution of bioeconomy produced energy for fossil based energy, there are two factors which mitigate impacts on land based biomass production. First, a change in policy orientation will lead to a greater efficiency in the use of available bioresources (waste and residuals) and second, a lower rate of economic growth will reduce total energy demand and subsequent pressure on biomass production as an input. Agricultural intensification has resulted in loss of habitat heterogeneity. It has been suggested that recreating landscape heterogeneity is “the key to restoring and sustaining biodiversity in temperate agricultural systems” (Benton et al. 2003). In a future with a policy orientation centered on sustainable development (SSP1), we see a possible shift towards more diverse agricultural systems that focus on multifunctional landscapes (NBP1) providing not only provisioning ecosystem services but also regulating services including pest control (Rusch et al. 2016). In addition, a higher level of societal environmental concerns would reduce the amount of land used for animal production. In both forestry and agriculture, reductions in production intensity will allow land to be set aside in environmentally sensitive areas.

SSP1 also includes both technological innovation and allocation of the needed human and financial resources (O’Neill et al. 2017). Developments in this SSP will be driven by a higher priority for environmental protection resulting from a change in attitudes. However, these conclusions pertain to global developments. For the Nordic countries, a change in attitudes may coincide with the requisite technological innovation irrespective of developments in other parts of the world. Though the results of SSP1 may appear to be similar to the fragmented world (SSP3 and NBP3), the difference here is openness to trade. Nordic innovations to reduce the use of fossil fuels would lead to exports of this technology and spur further movement along this pathway.

### NBP2: Conventional first—Don’t rock the boat (Box 2)

This pathway is an extension of current trends, including the current trend in the Nordic countries towards greater sustainability. For example, current policy in Denmark aims for fossil independence by the year 2050 (IEA 2017). While this would lead to an increase in bioenergy as a share of total energy use, significant technological breakthroughs of the type identified in NBP1 would not occur on this pathway. This pathway assumes that current policy would not shift significantly but would follow institutional patterns already established for allocation of human and financial resources.

### NBP3: Self-sufficiency first—Building walls (Box 3)

This pathway seemed less plausible before the Covid-19 pandemic started in 2020. The interruption of trade and border closures enacted to slow the spread of the virus have shown how quickly the global system can fragment. Depending on the long-term measures taken this may also lead to a regional fragmentation of the type described in this NBP. One of the consequences that the current response has made apparent is the decrease in economic activity (GDP) associated with fragmentation. The reduction in economic activity has also resulted in a significantly lower demand for energy. In NBP3, lower demand for energy can be met with current resources including fossil fuel production (Norway) that is now primarily consumed only regionally in addition to regionally produced hydroelectric, wind and bioenergy power. The availability of these energy sources and the lower demand from reduced economic activity would not lead to any significant increase in energy production from the bioeconomy sector. In a de-globalizing future scenario that focuses on regional security, technology development in agriculture is low and trade is restricted (SSP3) (O’Neill et al. 2017). In a Nordic context we envisioned that crop production is expanded where possible with conventional input intensive systems to increase self-sufficiency.

### NBP4: City first—Maintaining the divide (Box 4)

This pathway is the most difficult one to associate with impacts on the bioeconomy sector. While NBP4 is increasingly plausible as a high percentage of the population in the Nordic countries already lives in cities, there is uncertainty about how a small elite population would regard environmental policies. Even if there were islands of high environmental quality that served this elite it would be difficult to isolate these islands from surrounding environmental impacts. A resource-strong elite could assign a greater priority to bioresources that they controlled and in an open economy this would lead to some specialization in trade between countries. Furthermore, a large population dependent on limited resources would not be expected to prioritize regional environmental issues, this could lead to a greater dependence on locally produced energy.

### NBP5: Growth first—Running on the treadmill (Box 5)

In NBP5, the economic forces driven by open market competition would lead to specialization and greater returns on available resources. Greater returns may be expected to result in consolidation of production into larger units. However, due to biophysical limitations this would not lead to shifts in land use but would promote a high degree of resource use intensity as owners of resources compete for access to markets by lowering production costs. In the fossil-fuelled development (SSP5), international trade is high, with regional specialization in production and an environmental policy focused on the local environment, addressing issues with obvious benefits to well-being. Agriculture is highly managed and resource-intensive (O’Neill et al. 2017). This was interpreted as intensification of monocultures (NBP5), because these systems require resources in the form of agrochemicals to maintain productivity and provide pest control. While biodiversity consideration and loss of regulating services was not considered to have “obvious benefits to wellbeing”.

## Discussion

There have been two guiding principles in the development of this work:

- New regional narratives should be consistent with existing global scenarios.
- Narratives should support creation of parameter inputs for high resolution, catchment-scale, process-based models to evaluate the impact of changes in land management on delivery of water-related ecosystem services.

In futures studies, the choice of whether to use a top-down or bottom-up approach can be driven by a number of factors and leads to different tradeoffs. Using a bottom-up, stakeholder-driven approach could have simplified the process of identifying possible futures for the Nordic land-based bioeconomy but would have run the risk of lacking global context. The top-down approach we followed ensured our results could be put into a global context but also highlighted the difficulties of using generalized, global scenarios for framing regional, sectoral futures. From a global perspective, the Nordic countries are socially, economically and biophysically homogeneous. However, from a Nordic perspective, there are important differences. Each country has different cultures, different economies, and markedly different agricultural and forest sectors. There can also be significant differences in agricultural and forestry policies and practices within the individual countries.

Popp et al. (2017) quantified baseline SSP storylines and mitigation versions designed to reach several RCP target forcing levels. The quantified baseline SSPs cover a wide range of future land use and land cover changes. SSP1 has one of the lowest demands for agricultural goods (in 2100) combined with high intensification of agricultural production, which leads to the steepest decrease in agricultural land areas, and highest increase in forestland. On the other end, an increasing global population combined with low agricultural intensification leads to the greatest increase in agricultural land area for SSP3, while the forest area declines most (Popp et al. 2017). These results portray changes at the global scale, at which there are critical areas in need of sustainable agricultural intensification (Rockstrom et al. 2017; Scherer et al. 2018). In the Nordic countries, agricultural land is already intensively managed (Pradhan et al. 2017). In view of this, we interpreted the qualitatively described trends in SSP1, e.g. “improvements in agricultural productivity and rapid diffusion of best practices” together with policy orientation “towards sustainable development” (O’Neill et al. 2017) as a shift towards more diverse agricultural landscapes with a focus on provision of multiple ecosystem services.

While land cover is included in the SSPs, land management practices are not, which is important given the 2050 time horizon of the NBPs. Using 2050 as a time horizon was already determined in the BIOWATER project design. Although this time horizon does not encompass any significant changes in climate, it is appropriate for the focus on changes in land management at the core of the BIOWATER project. From an agricultural perspective, production decisions such as crop choices and cultivation practices are most often made with a short (< 5 year) horizon. Additionally, since changes in forest area in the Nordic countries are limited by biophysical constraints increasing biomass production and harvest in response to bioeconomy development are mainly associated with changes in short horizon management practices rather than long horizon land cover choices. While differences in the SSP and NBP time horizons are likely to result in dissimilar final conditions, the SSPs also describe trajectories. In developing the NBP storylines the SSP database (https://tntcat.iiasa.ac.at/SspDb) was used to ensure that the expected NBP conditions were consistent with estimates of key socio-economic in the SSP database for 2050.

The methodology that we used for extending the SSPs resembles recent methods used by other research groups for regional and/or sectoral extensions (Mitter et al. 2019; Kok et al. 2019; Zandersen et al. 2019). However, the present study differed from these in several important respects. In accordance with previous studies, the global SSPs provided the boundary conditions for our narrative development process. The process documented here evolved from a need for credible and scientifically robust local (catchment-scale) scenarios related to the future bioeconomy. SSP elements without a direct impact on the land-based bioeconomy were not explicitly considered. In accordance with how the SSPs were developed (i.e., without climate induced socioeconomic changes), the NBP trends are not a result of climate change, but purely driven by societal changes. It is thus possible to combine the NBPs with a range of future climates, e.g., the representative concentration pathways (RCPs) (van Vuuren et al. 2011), in subsequent modelling work.

The NBP storylines are not definitive. They are sketches of plausible futures that contain key words which can lead to creative interpretation by modellers and regional stakeholders to describe land management changes to be simulated in process based models. We must emphasize that the NBP storylines do not provide answers but rather provide a framework for evaluating changes based on plausible futures consistent with global and regional developments.

With the results in hand, it is useful to reflect on the problems we encountered in the development process when the choice was made to adapt the SSPs as the basis for the NBPs. In addition to inconsistencies in time horizons between the global and regional scales, a more serious problem was that the SSPs were developed to focus on two dimensions: climate change adaptation and mitigation. The back-casting approach to SSP development ensured that there would be a spread in these two dimensions for the five storylines. In the NBPs we tried to follow a back-casting approach but did not define the dimensions independently and instead worked with the same two SSP dimensions. This led to difficulties in justifications for element mapping and the subsequent storylines with respect to the role of bioeconomy development. Perhaps this could have been alleviated by defining dimensions more specific to the problem being addressed.

## Conclusions

For the Nordic countries and other small, open economies, future trends are not independent from global developments. Although there are drawbacks to adapting well-established global future studies such as the SSPs for regional and sectoral purposes, there are both associated efficiencies and advantages. Efficiencies are primarily a result of being able to use the significant amount of research that went into SSP development and the growing body of related literature. The advantages are that not only did using the SSPs allow for the required rapid scenario development to use in planned BIOWATER research, the NBP development process demonstrates a method for downscaling a global narrative to increase the relevance of scenario work in high resolution process-based models.

Within the Nordic countries, it is important that local (catchment) scale land management scenarios are consistent with regional trends. The NBPs provide qualitative narratives that have already been used in a series of national stakeholder workshops in each of the Nordic countries for transforming the storylines into quantitative values (Stages II and III in Fig. 1). These values will in turn be used in BIOWATER for modelling impacts on delivery of water-related ecosystem services in selected small Nordic catchments. Outputs from these studies can support Nordic policymakers when making decisions related to the land-based bioeconomy and the WFD.
